# Mechanisms Underlying Dopamine-Induced Risky Choice in Parkinson’s Disease With and Without Depression (History)

**DOI:** 10.1162/CPSY_a_00011

**Published:** 2018-02-01

**Authors:** Monique H. M. Timmer, Guillaume Sescousse, Rianne A. J. Esselink, Payam Piray, Roshan Cools

**Affiliations:** 1 Donders Institute for Brain, Cognition and Behaviour, Centre for Cognitive Neuroimaging, Radboud University, Nijmegen, the Netherlands; 2 Department of Neurology, Radboud University Medical Centre, and Parkinson Centre Nijmegen, Nijmegen, the Netherlands; 3 Department of Psychiatry, Radboud University Medical Centre, Nijmegen, the Netherlands

**Keywords:** Parkinson’s disease, depression, risky choice, dopamine, loss aversion, prospect theory

## Abstract

Patients with Parkinson’s disease (PD) are often treated with dopaminergic medication. Dopaminergic medication is known to improve both motor and certain nonmotor symptoms, such as depression. However, it can contribute to behavioral impairment, for example, by enhancing risky choice. Here we characterize the computational mechanisms that contribute to dopamine-induced changes in risky choice in PD patients with and without a depression (history). We adopt a clinical–neuroeconomic approach to investigate the effects of dopaminergic medication on specific components of risky choice in PD. Twenty-three healthy controls, 21 PD patients with a depression (history), and 22 nondepressed PD patients were assessed using a well-established risky choice paradigm. Patients were tested twice: once after taking their normal dopaminergic medication and once after withdrawal of their medication. Dopaminergic medication increased a value-independent gambling propensity in nondepressed PD patients, while leaving loss aversion unaffected. By contrast, dopaminergic medication effects on loss aversion were associated with current depression severity and with drug effects on depression scores. The present findings demonstrate that dopaminergic medication increases a value-independent gambling bias in nondepressed PD patients. Moreover, the current study raises the hypothesis that dopamine-induced reductions in loss aversion might underlie previously observed comorbidity between depression and medication-related side effects in PD, such as impulse control disorder.

## INTRODUCTION

Parkinson’s disease (PD) is a neurodegenerative disorder characterized by, among other things, degeneration of dopaminergic neurons leading to striatal dopamine depletion. PD patients exhibit motor and certain nonmotor symptoms, which can be alleviated (to some extent) by dopaminergic medication. However, the effects of dopaminergic medication on cognitive and decision functions are more complex (Robbins & Cools, 2014).

Work with experimental animals, healthy volunteers, and PD patients revealed that dopaminergic medication enhances risky choice (Brand et al., 2004; Cools, Barker, Sahakian, & Robbins, 2003; Euteneuer et al., 2009; Rutledge, Skandali, Dayan, & Dolan, 2015; St. Onge, Chiu, & Floresco, 2010; St. Onge & Floresco, 2009). In PD, these side effects can contribute to severe psychiatric abnormalities, including drug and gambling addiction (Weintraub et al., 2010). However, both the nature and extent of these psychiatric abnormalities vary greatly across patients. For example, although all patients receive dopaminergic medication, impulse control disorder (ICD) occurs only in a subset (∼ 10*%* − 15*%*; Weintraub et al., 2010). In keeping with this clinical variability, we know that there is also large individual variability in the nature and extent of dopaminergic drug effects on cognitive and decision functions (e.g., Cools & D’Esposito, 2011). Here we aimed to characterize the mechanisms that contribute to individual variability in medication-induced changes in risky choice in PD. To this end, we adopted a controlled medication withdrawal procedure to assess effects of dopaminergic medication on a well-established risky choice paradigm in PD.

We asked two specific questions, which were inspired by a number of apparently discrepant observations. First, we asked whether the extent to which dopaminergic medication increases risky choice in PD depends on the presence of depression (history), a frequent nonmotor symptom of PD. This question was raised by clinical observations that ICDs are often comorbid with depression (Joutsa, Martikainen, Vahlberg, Voon, & Kaasinen, 2012; Voon, Sohr et al., 2011). Based on this literature, dopamine-induced increases in risky choice might be expected to be greater in PD patients with than without a depression (history).

This prediction, however, contrasts with an alternative hypothesis, derived from the dopamine overdose account, which states that dopamine-induced deficits reflect detrimental overdosing of dopamine levels in relatively unaffected brain regions, such as the ventral striatum (Cools, 2006; Cools, Barker, Sahakian, & Robbins, 2001; Swainson et al., 2000). Based on evidence that depression in PD is accompanied by disproportionately reduced ventral striatal dopamine (Remy, Doder, Lees, Turjanski, & Brooks, 2005; Vriend et al., 2013; Weintraub et al., 2005), we might predict dopamine-induced increases in risky choice to be greater in nondepressed than in depressed PD patients. Our design allowed us to disentangle these two contrasting hypotheses.

Our second question was which computational mechanisms contribute to drug-induced increases in risky choice. To decompose drug effects on risky choice, we employed a computational modeling approach based on prospect theory, one of the more successful accounts of decision making under risk (Kahneman & Tversky, 1979,s). Unlike model-free analyses of choice patterns, this model-based approach enabled us to disentangle two different mechanistic hypotheses regarding the nature of drug effects.

The first mechanistic hypothesis was inspired by recent theorizing and empirical evidence indicating a key role for striatal dopamine in the relative weighting of reward versus punishment on learning and choice (Collins & Frank, 2014). Thus one mechanism by which dopaminergic medication may increase risky choice is by attenuating loss aversion, which reflects our tendency to weigh losses more than equally sized gains. Loss aversion is one of the core concepts of prospect theory (Kahneman & Tversky, 1984, 1979). In the domain of learning, studies with healthy volunteers and PD patients have demonstrated repeatedly that the balance between learning from reward and punishment depends critically on striatal dopamine (Cools, Altamirano, & D’Esposito, 2006; Frank, Seeberger, & O’Reilly, 2004; van der Schaaf et al., 2014). Increases in dopamine enhance reward-based relative to punishment-based learning, while decreases in dopamine enhance punishment-based relative to reward-based learning. Recent theoretical work extends these value-dependent effects from learning to choice (Collins & Frank, 2014), and empirical evidence has indeed demonstrated that dopami nergic medication in PD alters reward- versus punishment-based choice in ways very similar to its effects on learning (Shiner et al., 2012; Smittenaar et al., 2012). Here we aimed to investigate whether dopaminergic medication alters risky choice in PD in an analogous manner, by increasing the relative weighting of rewards (gains) versus punishments (losses), thereby attenuating loss aversion.

A second mechanism by which dopaminergic medication might affect risky choice is by altering the tendency to gamble in a value-independent manner. This is based on recent studies with healthy volunteers revealing that levodopa increases risky choice by increasing the propensity to gamble regardless of the gamble values at stake (Rigoli et al., 2016; Rutledge, Skandali, Dayan, & Dolan, 2015).

To address our two specific questions, we adopted a two-step approach. First, we assessed whether dopamine-induced increases in risky choice, as observed previously in nondepressed PD (Brand et al., 2004; Cools et al., 2006; Euteneuer et al., 2009), can best be accounted for by decreases in loss aversion and/or increases in a value-independent gambling bias. To this end, we assessed medication effects on prospect theory–derived parameters, representing loss aversion and gambling bias, from choice patterns on the risky choice task obtained from a psychiatrically clean group of PD patients without depression. Second, we assessed whether effects of dopaminergic medication on risky choice computations vary as a function of (individual differences in the current severity of) depression.

## METHODS

### Participants and Experimental Design

We recruited 23 nondepressed PD patients, 24 PD patients with a depression (history), and 25 healthy controls. Data from one nondepressed PD patient, three PD patients with a depression (history), and two healthy controls were discarded from the analyses (see “Exclusion”). The final analysis included 22 nondepressed PD patients, 21 PD patients with a depression (history), and 23 healthy controls.

Patients were recruited from the Parkinson Centre at the Radboud University Medical Centre, the Netherlands. Healthy controls were recruited via advertisement or were partners or acquaintances of patients. Healthy controls and patients were matched for gender, age, and IQ measured with the Dutch version of the National Adult Reading Test (NART; Schmand, Bakker, Saan, & Louman, 1991). Patient groups were matched in terms of disease severity, measured with the Unified Parkinson’s Disease Rating Scale, Part III (UPDRS–III; Goetz & Stebbins, 2004) and used similar amounts of dopaminergic medication, levodopa equivalent dose (LED; Esselink et al., 2004; Table 1). Written informed consent according to the Declaration of Helsinki was obtained from all participants. The study was part of a larger project investigating the neurobiological mechanisms of depression in PD and was approved by the local ethics committee (Commissie Mensgebonden Onderzoek regio Arnhem–Nijmegen, the Netherlands, nr. 2012/43).

**Table 1.** Group characteristics

	**PD with a depression (history) *n* = 21**	**Nondepressed PD *n* = 22**	**Healthy controls *n* = 23**
Gender, men	13	13	14
Age (years)	58.5 (5.8)	61.0 (7.6)	60.9 (5.9)
NART-IQ	96.2 (11.6)	97.0 (15.5)	100.7 (13.7)
MMSE	28.5 (1.4)	28.6 (1.3)	28.8 (1.2)
Hoehn & Yahr	1.6 (0.4)	1.8 (0.5)	–
UPDRSIII (OFF)	22.7 (9.6)	22.2 (6.5)	–
Disease duration (years)	5.1 (3.5)	4.5 (2.2)	–
LED (mg/day)	551 (248)	627 (275)	–
LED agonists (mg/day)	71 (122)	103 (129)	–
BDI (OFF)	9.9 (6.1)	4.0 (2.3)	3.1 (2.1)
Current ICD	4	1	–
First session ON	11	9	–
Days between sessions	23 (27)	21 (20)	–
Endowment OFF session	11.20 (1.08)	10.52 (1.69)	11.36 (1.76)
Endowment ON session	11.28 (1.25)	11.26 (1.42)	–

*Note*. Values represent numbers or mean (standard deviation).

All patients were diagnosed with idiopathic PD according to the U.K. Brain Bank criteria (Gibb & Lees, 1988) by a neurologist specialized in movement disorders (Prof. B. R. Bloem, Dr. R. A. Esselink, Dr. B. Post) and were treated with dopaminergic medication. In the non depressed group, 11 patients were treated with levodopa, 2 were treated with dopamine receptor agonists, and 9 were treated with both. In the group of PD patients with a depression (history), 14 patients were treated with levodopa, 2 were treated with dopamine receptor agonists, and 5 were treated with both. Patients were on stable medication regimes during the course of the study, except for one patient, who used duloxetine—a serotonin/noradrenalin reuptake inhibitor prescribed to treat pain—for 4 weeks between the 2 testing days (in this case, testing days were separated by 17 weeks). The drug was discontinued 4 weeks before the second testing day. Seven patients in the patient group with a depression (history) received antidepressants (paroxetine, *n* = 3; escitalopram, *n* = 1; venlafaxine, *n* = 1; and nortriptyline, *n* = 2).

Patients were included in the depression (history) group if they met the DSM-IV criteria for any of the various depression-related diagnoses in the DSM-IV within a timeframe of 5 years before PD diagnosis up until the present. This 5-year cutoff was chosen because the incidence of depression is significantly higher within the 5 years before PD diagnosis (Shiba et al., 2000). These depressive episodes are therefore more likely related to PD pathology. Seven patients were diagnosed with a major depressive episode (3 past, 4 current), 12 with a minor depressive episode (9 past, 3 current), 1 with a past dysthymic disorder, and 1 with a past adjustment disorder with depressed mood. Thus note that the patient group with a depression (history) consisted of patients with both past (*n* = 14) and current (*n* = 7) depression. This heterogeneity allowed us to specifically assess dopaminergic medication effects on risky choice parameters as a function of current depression severity. Indeed, only seven patients were identified as being depressed at the time of testing. As such, any conclusions regarding the effects of depression across the group of patients with depression (history) should be interpreted as reflecting effects of either current or past depression. Psychiatric diagnosis was based on structured psychiatric interviews administered during an intake session (MINI-plus; Sheehan et al., 1998). General exclusion criteria were clinical dementia (Mini Mental State Examination [MMSE] < 24; Folstein, Folstein, & McHugh, 1975), psychiatric disorders other than depression (bipolar disorder, schizophrenia, attention-deficit hyperactivity disorder, or drug or alcohol abuse), neurological comorbidity, and hallucinations. Healthy controls were also excluded if they had a history of mood or anxiety disorder or a history of obsessive-compulsive disorder or if they used any psychotropic medication.

Patients were assessed on two occasions: once after taking their normal dopaminergic medication (ON) and once after abstaining from their dopaminergic medication for at least 18 hours (24 hours for slow-release dopamine receptor agonists; OFF). Antidepressants were taken on both testing days, enabling us to assess specifically dopaminergic medication effects on gambling behavior. The order of ON and OFF sessions was counterbalanced in each patient group (Table 1). Healthy controls were only tested once. During testing sessions, we administered the gambling task described herein. Furthermore, on each testing day, participants completed the Beck Depression Inventory (BDI; Beck, Erbaugh, Ward, Mock, & Mendelsohn, 1961) to assess current depressive symptoms. Participants were instructed to answer BDI questions according to how they felt over the past 24 hours (rather than the past week), enabling us to assess dopaminergic drug (withdrawal) effects on depression scores. Patients also completed the Questionnaire for Impulsive-Compulsive Disorders in Parkinson’s Disease-Rating Scale (QUIP-RS; Weintraub et al., 2012) developed to assess ICD symptoms in PD, and the UPDRS–III was administered to assess clinical motor symptom severity (Goetz & Stebbins, 2004).

Participants were paid a fixed amount per testing day for participation (healthy controls, €30; patients, €40) and received an additional amount of money based on task performance (between €2 and €11 per session; see later).

### Task

**Figure 1.**
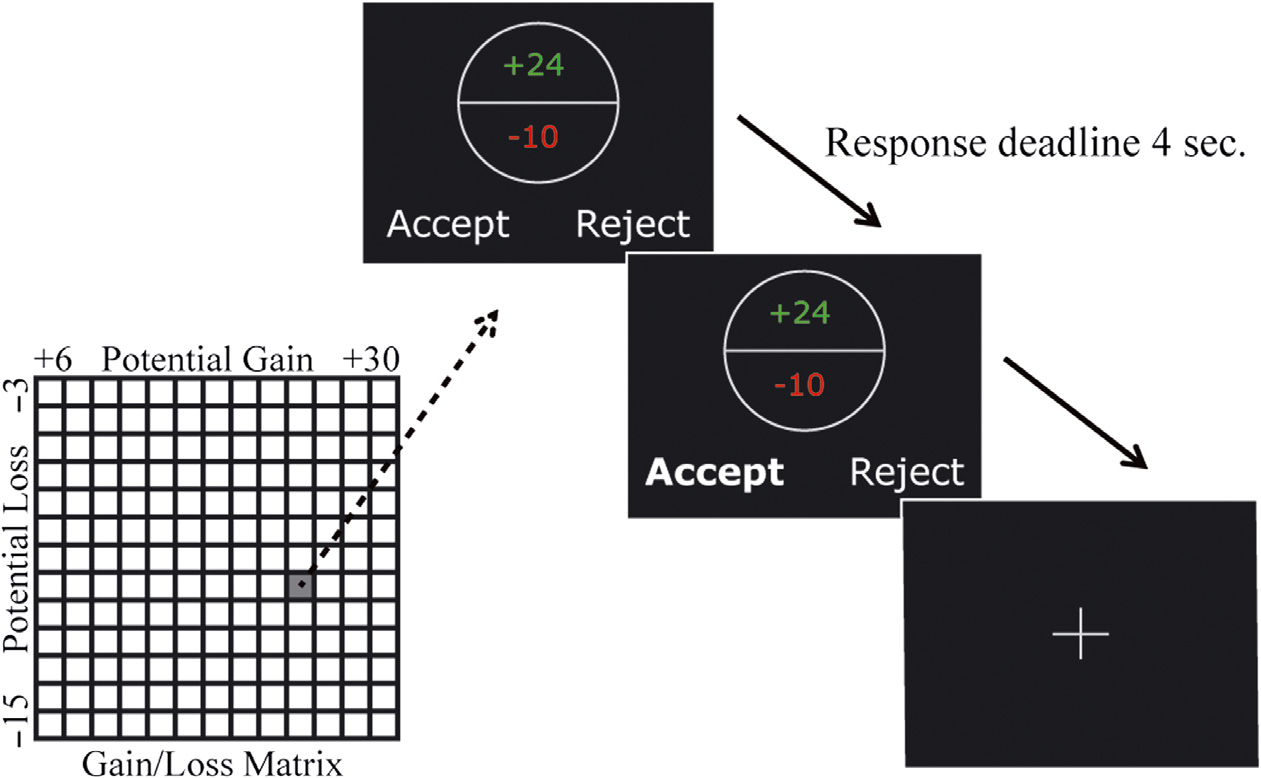
**Task overview.** Participants played a gambling task designed to measure loss aversion. During this task, participants were presented with 169 mixed gambles, each offering a 50/50 chance of either gaining or losing varying amounts of money. Gains ranged from +€6 to +€30 (increments of €2); losses ranged from −€3 to −€15 (increments of €1; see gainloss matrix). Each possible gainloss pair was presented once in randomized order. Participants were asked either to accept (play) or reject the gamble within a maximum time of 4 s.

Participants played a well-validated gambling task designed to measure loss aversion (Tom, Fox, Trepel, & Poldrack, 2007; Figure 1). During this task, participants were presented with 169 mixed gambles (split into three runs) on a computer screen. Each gamble offered a 50 − 50 chance of either gaining or losing varying amounts of money. Potential gains ranged from +€6 to +€30 (increments of €2), while potential losses ranged from −€3 to −€15 (increments of €1). This asymmetric gain–loss range was chosen to maximize statistical power, based on the assumption that on average, people are twice as sensitive to losses as they are to gains (Tom et al., 2007). Each of the possible gain–loss pairs (13 × 13 = 169) was presented once in randomized order. Participants were asked either to accept (play) or to reject the gamble by pressing one of two buttons. To make participants feel that they were gambling with *their own money*, and thus avoid “house money effects” (Thaler & Johnson, 1990), endowments at the beginning of this gambling task were earnings from a behavioral experiment immediately preceding the present experiment on the same day. Gambles were not resolved during the experiment to exclude trial-by-trial behavioral adjustments based on previous earnings. However, to ensure that participants would take each gamble seriously, at the end of the experiment, three gambles were randomly selected and played for real money.

## ANALYSIS

### Model

We used a model-based approach to analyze participants’ choice behavior. This procedure involved fitting a theoretical model of decision making to behavioral data to quantify specific aspects of choice behavior. One of the most popular accounts of decision making under risk is prospect theory (Kahneman & Tversky, 1979). We sought to understand the effects of dopaminergic medication and depression in PD by assessing how these factors modulate the parameters obtained from a model based on prospect theory. Within that framework, the subjective utility of each gamble (SUG) can be approximated by the following equation:

$$\text{SUG}=p_{\text{Gain}}\times \text{Gain}-p_{\text{Loss}}\times \text{Loss}\times \lambda ,$$

where *p*_Gain_ is the gain probability, *p*_Loss_ is the loss probability, Gain is the gain value of the gamble, and Loss is the (absolute) loss value of the gamble. The relative weighting of gains and losses is reflected in the loss aversion parameter *λ*. If *λ* > 1, then losses are overvalued relative to gains: A person is loss averse. If *λ* < 1, then gains are overvalued relative to losses: A person is loss seeking. If *λ* = 1, gains and losses are valued equally: A person is gain–loss neutral.

A softmax function was used to estimate the probability of gamble acceptance based on the subjective value of the gamble:

$$p\left(\text{gamble}\;\text{acceptance}\right)=\frac{1}{1+e^{-\mu \left(\text{SUG}+\text{c}\right)}}.$$

This function includes two other parameters; an inverse temperature parameter (μ) and a constant parameter (c). The constant parameter (c) reflects a value-independent gambling bias toward or away from gambling. If c > 0, there is a tendency to accept gambles regardless of their subjective utility. If c < 0, there is a tendency to reject gambles regardless of their subjective utility. The inverse temperature parameter reflects consistency of choice behavior. If μ = 0, choices are random, whereas if μ is highly positive or negative, there is consistency in choice behavior, with a positive μ representing higher gamble acceptance with higher gain and lower loss value (and vice versa for negative μ). Model parameters were constrained as follows: From 0 to 10 for the loss aversion parameter (*λ*) and the inverse temperature parameter (μ) and from − 10 to 10 for the value-independent gambling bias parameter (c). We anticipated μ to be positive, consistent with a utility maximization strategy, where participants accept more gambles when gain values increase and loss values decrease. None of the parameters obtained from our participants reached these boundaries (except for the two patients who were excluded from the analysis; see “Exclusion”).

The model that we fitted to the data assumes a linear valuation of gains and losses, in contrast to the curvilinear value function of prospect theory. This is a common and reasonable simplifying assumption given the relatively narrow range of gains and losses used in this protocol. We also assumed no subjective transformation of probabilities as described in prospect theory and thus assumed equal weights for the 0.5 probability of gains and losses (De Martino, Camerer, & Adolphs, 2010; Tom et al., 2007).

### Exclusion

We assessed whether participants’ choices were influenced by gain and loss values in an expected manner, that is, whether participants were utility maximizers (accepting more gambles with increasing gain values and accepting fewer gambles with increasing loss values). Inspection of the individual response patterns revealed that two participants (one patient with a depression [history] and one nondepressed patient) did not meet this a priori assumption, suggesting a lack of understanding of task instructions. In both cases, this was during the first testing day. In one case, the response pattern revealed that the participant accepted more gambles when gain values decreased and loss values increased, thereby unintentionally trying to minimize earnings. During debriefing, this participant realized that he had made a mistake. The responses of the other participant were suggestive of random choice behavior. In both cases, these observations were confirmed by negative temperature parameters (μ) obtained from the model. These two patients were excluded from further analyses. Moreover, two healthy controls were excluded from further analyses because of a lifetime history of depression, while two PD patients with a depression (history) were excluded because they failed to finish the study, leading to incomplete datasets.

### Model Fitting and Comparison

We used a hierarchical Bayesian fitting procedure to fit the model to participants’ choices as described by Huys and colleagues (Huys et al., 2011; Huys et al., 2012). This method estimates the mean and variance of model parameters across all subjects and sessions. These parameters then serve to define a normal a priori distribution for finding individual values of parameters for each subject and session (i.e., posterior parameters). We hypothesized the a priori distributions of the relevant parameters (the loss aversion parameter, *λ*, and the gambling bias parameter, c) to be different for patients and healthy controls. Therefore we first fitted the model to patient data only. Note that any differences in posterior parameters between patient groups and medication sessions cannot be attributed to parameter regularization employed during fitting, because individual parameters from both patient groups and both drug sessions were obtained using the same a priori distribution (Huys et al., 2012). To compare PD patients with healthy controls, we fitted the model to healthy control and patient data together (separately for each drug session).

The hierarchical Bayesian fitting procedure is an iterative algorithm. In every iteration, individual parameters are optimized based on the data and current estimation of the group mean and variance. We used a Laplace approximation for defining a normal approximation of individual posteriors, in which the maximum a posteriori values were found using nonlinear optimization methods (MATLAB optimization toolbox, fmincon routine, interior-point algorithm). The group mean and variance are then updated according to obtained individual posteriors (for equations, see Huys et al., 2012), which serve as group mean and variance in the next iteration. The iterative updating of group and individual parameters continues until changes in parameters are very small (i.e., a convergence criterion satisfied). Importantly, convergence is guaranteed in this algorithm (Bishop, 2006).

A Bayesian model comparison was conducted to compare the model with three parameters (*λ*, μ, and c) with a slightly simpler model, where we forced c to be zero, thereby reducing the number of free parameters. This model assumed that subjects do not exhibit a value-independent bias toward or away from gambling. A Bayesian model comparison assessed which model best captured participants’ choices by computing model evidence by balancing model fits and model complexity (Kass & Raftery, 1995; Piray et al., 2014). A procedure was employed that penalizes complexity by marginalizing over both group and individual parameters using Laplace approximation and the Bayesian information criterion, respectively. The negative log-mode evidence (NLME) was computed as

$$\text{NLME}\approx -\underset{n}{\sum }\log P(D^{n}{|\theta }^{n})-\underset{n}{\sum }\log N{(\theta }^{n}|\Theta ,\Sigma )-\frac{1}{2}mN\log2\pi +\frac{1}{2}\underset{n}{\sum }\log|H_{n}|+m\log(N),$$

where *D*^*n*^ is the set of choice data for the *n*th participant; θ^*n*^ is the fitted individual parameter for the *n*th participant; *Θ* and *Σ* are the mean and variance for the group distribution, respectively; *m* is the number of free parameters of the model; *N* is the number of participants; and |*H*_*n*_| is the determinant of the Hessian matrix of the log-posterior function at θ^*n*^. The first term on the right-hand side of the equation refers to how well the model predicts data. The sum of the next three terms together is the penalty due to individual parameters. The last term represents the penalty approximated for 2*m* (mean and variance together) group parameters using the Bayesian information criterion (Piray et al., 2014). The model with the lowest NLME is the best model.

### Statistical Analysis

First, we examined whether dopaminergic medication modulated loss aversion and/or gambling bias parameters in nondepressed PD patients. Subsequently, we compared medication effects on these parameters between nondepressed PD patients and PD patients with a depression (history). Finally, we compared patients’ data with those of age-matched controls, each group and drug session separately. Because loss aversion and gambling bias parameters were nonnormally distributed (Shapiro-Wilk, *p* < 0.05), we used two-tailed Wilcoxon signed-rank tests to assess within-subject differences and Mann–Whitney tests to assess between-group differences. Depression scores and proportion of accepted gambles, which were normally distributed, were analyzed with a mixed analysis of variance (ANOVA) with drug as within-subject and group as between-subject factor. Two-tailed Pearson correlations were used for normally distributed data, and two-tailed Spearman correlations were used for nonnormally distributed data. Furthermore, for nonnormally distributed data, we reported medians and their standard errors. Standard errors of the median were computed using bootstrapping (Efron & Tibshirani, 1993). By resampling with replacement of the original group sample, we created 10^5^ new group samples. The standard error of the median was then defined as the standard deviation of all bootstrapped samples.

## RESULTS

### Risky Choice and Drug Effects in Nondepressed Parkinson’s Disease (PD) Patients

Patient and disease characteristics are presented in Table 1. Median parameters obtained from the model (per group and medication session) are presented in Table 2. Individual endowments at the beginning of the experiment varied between participants (Table 1), as these were earnings from a previous experiment performed the same day. However, there was no significant main effect of drug on earnings.

**Table 2.** Model parameters per group and drug session

	**OFF session**	**ON session**
**Gambling response bias (c)**		
PD with a depression (history)	−1.73 (14.9)	−1.30 (13.8)
Nondepressed PD	−2.71 (9.4)	−1.05 (8.9)
Healthy controls	−0.65 (11.1)	–
**Loss aversion (*λ*)**		
PD with a depression (history)	1.51 (3.0)	1.19 (2.7)
Nondepressed PD	1.01 (3.2)	1.16 (2.6)
Healthy controls	1.37 (2.8)	–
**Inverse temperature (*μ*)**		
PD with a depression (history)	0.93 (2.1)	0.94 (1.9)
Nondepressed PD	0.89 (1.5)	1.09 (2.2)
Healthy controls	1.06 (2.1)	–

*Note*. Values represent median (range).

Using prospect theory–based analysis, we assessed the computational mechanisms contributing to risky choice. The full model including a constant parameter (c; reflecting a gam bling response bias irrespective of the value of gambles) provided a better account of participants’ choices than did a model without this parameter, indicated by a lower log-model evidence (in patients, 4,102 compared with 4,374 for the model where c was forced to be zero; in healthy controls, 1,099 compared with 1,131 for the model where c was forced to be zero). Therefore reported results are based on parameters obtained from the full model. First, we assessed medication effects on model parameters derived from risky choice patterns in nondepressed PD patients.

**Figure 2.**
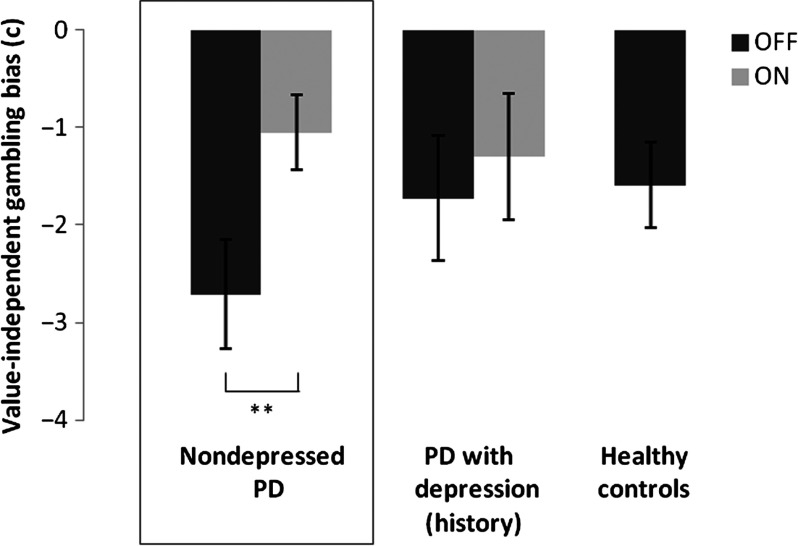
**Drug effects on value-independent gambling bias.** Median value-independent gambling bias parameter (*c*) per session (OFF session in dark gray; ON session in light gray) in nondepressed PD patients. For illustration purposes, we also added the bars for PD patients with a depression (history; OFF and ON sessions) and for healthy controls (OFF session). Error bars represent standard errors of the median. ^**^ *p* < 0.01.

Analysis of the value-independent gambling bias parameter (c) revealed negative parameters during both drug sessions (Table 2), indicating a tendency to avoid gambling irrespective of the value of the gambles. Dopaminergic medication significantly increased the value-independent gambling propensity in nondepressed PD patients, *Z* = −2.65, *p* = 0.008 (Figure 2). There was no effect of dopaminergic medication on loss aversion (*λ*), *Z* = −1.54, *p* = 0.12, and no effect on the inverse temperature parameter (μ), *Z* = −0.18, *p* = 0.86. Moreover, there were no effects of medication dose (LED) and no session order effects.

### Effect of Depression (History) in Parkinson’s Disease (PD)

Subsequently, we compared nondepressed PD patients and PD patients with a depression (history). Again, individual endowments at the beginning of the task varied between participants (Table 1). There was no significant main effect of Group or Drug and no Group × Drug interaction on these earnings.

Analyses of the value-independent gambling bias parameter (c) revealed a near-significant Group × Drug interaction, *U* = 156, *p* = 0.07, indicating that dopaminergic medication tended to increase a value-independent gambling propensity to a greater extent in nondepressed PD patients compared with PD patients with a depression (history). While the medication had a clear effect on value-independent gambling propensity in nondepressed PD patients, as reported previously, it had no effect in PD patients with a depression (history), *Z* = −0.087, *p* = 0.9. The main effect of Drug failed to reach significance, *Z* = −1.78, *p* = 0.08. There was no main effect of Group, *U* = 199, *p* = 0.44. There was no significant correlation between drug effects on value-independent gambling bias and current depression severity (BDI score).

**Figure 3.**
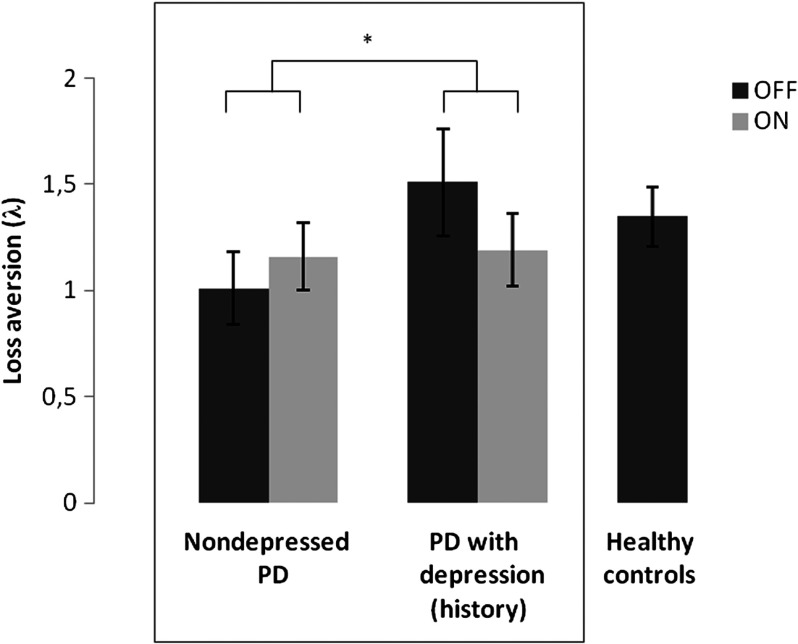
**Drug effects on loss aversion.** Median loss aversion parameter (*λ*) per group (non depressed PD patients and PD patients with a depression [history]) and drug session (OFF session in dark gray; ON session in light gray). For illustration purposes, we also added the bar for healthy controls (OFF session). Error bars represent standard errors of the median. *p < 0.05.

Analyses of the loss aversion parameter (*λ*) revealed a significant Group × Drug interaction, *U* = 149, *p* = 0.046. PD patients with a depression (history) exhibited greater drug-induced decreases in loss aversion than nondepressed PD patients did. However, the simple main effects of Drug were not significant. There was a near-significant simple main effect of Group in the OFF state; patients with a depression (history) tended to be more loss averse than nondepressed patients were, *U* = 151, *p* = 0.052. During the ON state, there was no simple main Group effect, *U* = 215, *p* = 0.70. There was no overall main effect of Group, *U* = 191, *p* = 0.33, and no overall main effect of Drug, *Z* = −0.21, *p* = 0.84 (Figure 3).

**Figure 4.**
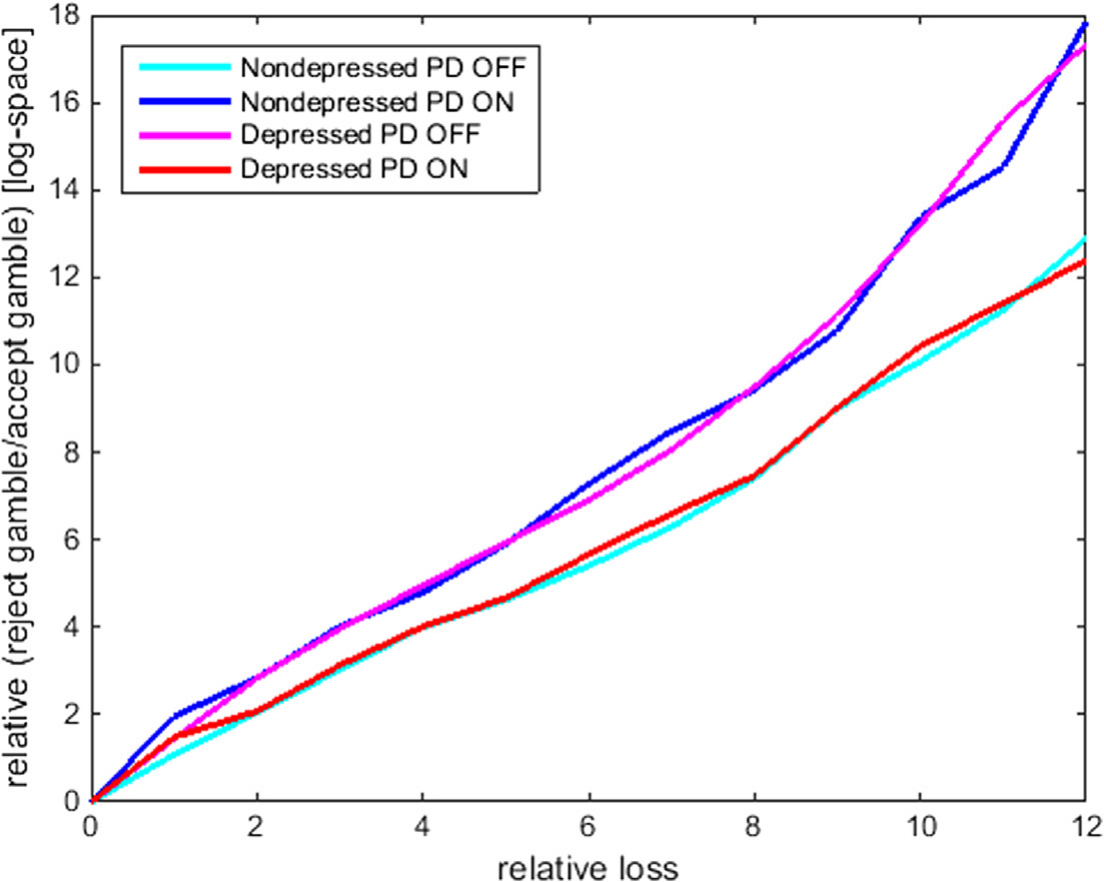
**Loss sensitivity.** The ratio of the number of rejected gambles divided by the number of accepted gambles in log-space (*y* axis) as a function of the relative loss averaged across different gain values (*x* axis) per group and per drug session. A steeper slope indicates greater loss sensitivity.

To visualize drug and group effects on loss aversion, we plotted, for each group and drug session separately, the degree to which the ratio of rejecting to accepting gambles increased as a function of increases in potential losses (raw data; Figure 4). To control for effects of other factors, such as general drug effects on gambling rate, we plotted the ratio of rejecting to accepting gambles as a function of relative loss differences between pairs of trials, while effects of different gains were averaged out. A steeper slope indicates greater loss sensitivity. From Figure 4, it is clear that dopaminergic medication had contrasting effects on loss aversion in nondepressed PD patients and in PD patients with a depression (history).

**Figure 5.**
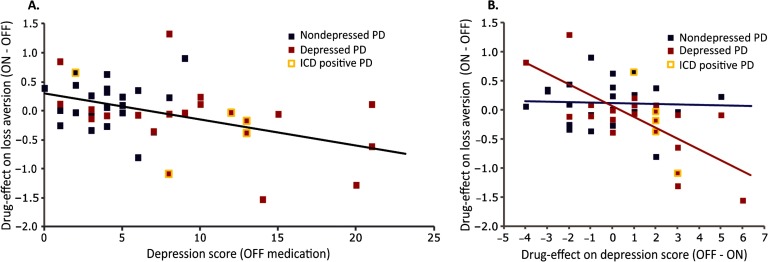
**Correlations between (drug effects on) loss aversion and depression.** A) Correlation between scores on the BDI during the OFF session (*x* axis) and drug effects on loss aversion (*λ*) on the *y* axis (ON session score minus OFF session score) across PD patients with and without a depression (history), *ρ*_(41)_ = −0.384, *p* = 0.011. B) Correlation between drug effects on depression scores on the *x* axis (BDI OFF session score minus ON session score) and drug effects on loss aversion (*λ*) on the *y* axis (ON session score minus OFF session score). Patients with a depression (history) are marked in red, *ρ*_(19)_ = −0.592, *p* = 0.005, and nondepressed patients are marked in blue, *ρ*(20) = −0.021, *p* = 0.93. This correlation was significantly different between groups (Fisher *r*-*z* transformation, *z* = −2.01, *p* = 0.044). Patients who screened positive for having an impulse control disorder are marked with a yellow border.

Medication effects on loss aversion were predicted by current OFF-state depression severity, *ρ*_(41)_ = −0.348, *p* = 0.022. This correlation was due to greater drug-induced decreases in loss aversion in patients with higher current OFF-state depression scores (Figure 5A). Moreover, drug effects on current depression scores correlated significantly with drug effects on loss aversion, *ρ*_(41)_ = −0.384, *p* = 0.011, indicating greater drug-induced decreases in loss aversion in patients with greater drug-induced decreases in depression scores. This correlation was strong in patients with a depression (history), *ρ*_(19)_ = −0.592, *p* = 0.005, but not significant in the nondepressed patients, *ρ*_(20)_ = −0.021, *p* = 0.93, and significantly different between groups (Fisher *r*-*z* transformation, *z* = −2.01, *p* = 0.044; Figure 5B).

There were no main effects of Drug, *Z* = −0.31, *p* = 0.75, or Group, *U* = 225, *p* = 0.88, and there was no significant Group × Drug interaction, *U* = 226, *p* = 0.90, on the inverse temperature parameter (μ). There were no effects of LED, no session order effects, and no effects of current ICD status on model parameters (value-independent gambling bias, loss aversion, and inverse temperature parameter).

Mixed ANOVA of depression scores (BDI) demonstrated a significant Group × Drug interaction, *F*(1,41) = 4.19, *p* = 0.047. Post hoc paired-sample *t* test revealed that this interaction was due to a significant drug-induced decrease in depression scores in patients with a depression (history), *t*(20) = 2.19, *p* = 0.041, but not in nondepressed patients, *t*(21) = −0.60, *p* = 0.56. There was also a main effect of Group, *F*(1,41) = 17.26, *p* < 0.001, indicating significantly higher depression scores in patients with a depression (history). There was no main effect of Drug.

Five patients exhibited at least one ICD as assessed with the QUIP-RS rating scale (one nondepressed patient and four patients with a depression [history]). None of them exhibited gambling addiction.

### Comparison with Healthy Controls

Relative to controls, nondepressed PD patients showed a significantly lower value-independent gambling bias during the OFF session, *U* = 111, *p* = 0.001, but not during the ON session, *U* = 177, *p* = 0.08. This is consistent with the significant effect of medication in the nondepressed PD group, mentioned earlier. Relative to controls, PD patients with a depression (history) showed a significantly lower value-independent gambling bias during the ON session, *U* = 151, *p* = 0.033, but not during the OFF session, *U* = 160, *p* = 0.06. Note however that, within this patient group, there was no significant medication effect on the value-independent gambling bias parameter.

Relative to controls, median loss aversion parameter estimates were quite low in patients. However, direct comparisons with controls revealed that this reduction was significant only in nondepressed PD patients OFF medication, *U* = 150, *p* = 0.019. The median loss aversion parameter from nondepressed patients ON medication, *U* = 169, *p* = 0.056, and from PD patients with a depression (history) OFF, *U* = 228, *p* = 0.75, or ON medication, *U* = 176, *p* = 0.12, did not differ from that of controls. There were no differences in terms of the inverse temperature parameter (μ) between controls and either group of PD patients (ON and OFF medication).

### Proportion of Accepted Gambles

In addition to the computational parameters underlying risky choice, we analyzed the proportion of accepted gambles, which is a compound measure of risky choice. The proportion of accepted gambles in PD patients with a depression (history) was 53.6% OFF medication and 57.9% ON medication. In nondepressed PD patients, this was 56.3% OFF medication and 60.2% ON medication. The proportion of accepted gambles in healthy controls was 62.2%. Mixed ANOVA in PD patients revealed no significant Group × Drug interaction, *F*(1,41) = 0.01, *p* = 0.94, and no main effect of Group, *F*(1,41) = 0.31, *p* = 0.58, or Drug, *F*(1,41) = 2.32, *p* = 0.136. The correlation between drug-induced increases in gamble acceptance and depression scores OFF medication failed to reach significance, *r*(41) = 0.273, *p* = 0.077. There was no significant correlation between LED and drug-induced increases in gamble acceptance, *r*(41) = 0.171, *p* = 0.27. Comparison of patients with healthy controls (each patient group and drug session separately) revealed no significant differences in gamble acceptance.

## DISCUSSION

The present study revealed two key findings. First, the data demonstrate that dopaminergic medication increases a value-independent gambling bias in nondepressed PD patients, as it does in healthy controls (Rigoli et al., 2016; Rutledge et al., 2015). This provides the third piece of converging evidence for dopamine-induced increases in gambling bias and reinforces the construct validity of this finding, also generalizing it across experimental paradigm and across underlying theoretical framework. Indeed, dopamine-induced increases in a value-independent gambling bias have now been shown using computational model-based analyses grounded in reinforcement learning theory (Rutledge et al., 2015), mean-variance theory (Rigoli et al., 2016), and prospect theory (Kahneman & Tversky, 1979). Second, the present data indicate that dopaminergic medication reduces loss aversion to a greater degree in PD patients with higher current depression ratings. The finding that dopamine modulates the relative weighting of gains versus losses during risky choice concurs generally with current theories about striatal dopamine’s role in valuation and choice (Collins & Frank, 2014) and raises the hypothesis, to be addressed in future studies, that dopamine-related reductions in loss aversion might underlie previously observed comorbidity between depression and medication-related side effects in PD, such as ICDs.

The present study illustrates the power of a computational model-based approach to analyze choice data. Indeed, the present study would have failed to reveal any of the effects on risky choice, if we had not taken into account the prior theoretical insight that the proportion of accepted gambles on tasks such as the one used here is a function of multiple parameters, including a value-independent gambling bias and loss aversion. Our observation raises the possibility that dopamine-induced increases in risky choice as measured previously using other tasks in nondepressed PD patients (i.e., the Cambridge Gamble Task and the Game of Dice task; Brand et al., 2004; Cools et al., 2003; Euteneuer et al., 2009) also reflect increases in a value-independent gambling bias.

Two previous studies (Rigoli et al., 2016; Rutledge et al., 2015) have reported dopamine-induced changes in a value-independent gambling bias. Both studies involved administration of an acute dose of levodopa to healthy volunteers, and both reported enhanced attraction to gambling for gains. Unlike Rutledge et al. (2015), we show that the gambling bias extends to gambles with mixed gains and losses, suggesting that the effect is not only value but also valence independent. As has been suggested previously (Friston et al., 2013; Kakade & Dayan, 2002; Rigoli et al., 2016; Rutledge et al., 2015), the nonspecific attraction to gambling might arise from an exploration bonus associated with surprising outcomes that potentiates information, sensation, and novelty seeking (Dagher & Robbins, 2009; Norbury, Manohar, Rogers, & Husain, 2013).

Based on the dopamine overdose hypothesis (Cools, 2006), we considered the hypothesis that dopamine’s effects on gambling (biases) might be greater in nondepressed PD patients, with putatively intact ventral striatal dopamine levels, than in patients with a depression (history), with putatively greater striatal dopamine deficiency. In fact, the comparison with controls suggests that nondepressed PD patients exhibit an abnormally reduced gambling bias when OFF medication rather than abnormally enhanced gambling bias when ON medication. This clearly is not consistent with the overdose hypothesis, although it should be noted that our design was not optimized for comparing patients with controls, who were tested only once in the absence of medication. Perhaps more importantly, although there was a statistical trend for a Group × Drug interaction, with marginally greater drug effects on value-independent gambling bias for nondepressed than (previously) depressed patients, this effect did not actually reach significance. Thus we provide no support for the hypothesis that medication effects on gambling (bias) are greatest in patients with less affected dopamine levels. Of course, the current study does not exclude the possibility that dopamine-induced increases in gambling bias are absent in PD patients with more severe current depression.

Dopaminergic medication decreased loss aversion to a greater extent in PD patients with a depression (history) than in nondepressed PD patients. However, the simple main effects of drug were nonsignificant. This could reflect the heterogeneity of our sample of depressed patients. Indeed, a weakness of the current study is that we included PD patients with present as well as past depression. However, it did allow us to investigate effects of current depression severity on mechanisms of risky choice. Correlation analyses revealed that dopamine-induced decreases in loss aversion were related to current depression severity and to effects of dopamine on depressive symptoms. Patients with the highest current depression scores and the greatest beneficial effect of dopaminergic medication on depression scores also exhibited the greatest dopamine-induced decrease in loss aversion. This finding concurs with clinical evidence indicating that PD patients who exhibit more severe depressive symptoms are at increased risk for having ICD (Joutsa et al., 2012), although a strong link between (dopamine-induced decreases in) loss aversion and ICD has yet to be established (Giorgetta et al., 2014; Voon, Gao et al., 2011).

The effect of medication on the gambling bias in the nondepressed PD patients represents a conceptual replication, and accordingly, we have considerable confidence in the reproducibility of the effect, also given that it remained significant after correcting for the multiple contrasts—(a) ON versus OFF in the nondepressed group and (b) nondepressed patients versus patients with a depression (history). Conversely, the effect of depression on loss aversion was marginally significant, particularly when taking into account the fact that multiple comparisons were conducted. Accordingly, we recommend that future studies aim to replicate this effect of depression on loss aversion.

To conclude, the present findings suggest that previously observed increases in risky choice in (nondepressed) PD patients on dopaminergic medication might reflect a value-independent change in a gambling bias. Moreover, the present study raises the hypothesis, to be addressed in future studies, that dopamine-induced reductions in loss aversion might underlie previously observed comorbidity between depression and medication-related side effects in PD, such as ICD.

## AUTHOR CONTRIBUTIONS

Monique H. M. Timmer, Guillaume Sescousse, Rianne A. J. Esselink, and Roshan Cools were responsible for the conception and design of the study. Monique H. M. Timmer, Guillaume Sescousse, Payam Piray, and Roshan Cools drafted and revised the report. Data were collected by Monique H. M. Timmer. Analyses were conducted by Monique H. M. Timmer, Guillaume Sescousse, and Payam Piray. All authors approved the final version of the manuscript.

## FUNDING INFORMATION

This project was funded by a grant from the “Stichting Parkinson Fonds,” Hoofddorp, the Netherlands. Roshan Cools was supported by an NWO Vici grant (453-14-015), and GS was supported by an NWO Veni grant (451-14-006).

## ACKNOWLEDGMENTS

We would like to thank all participants for their cooperation in the study.
